# Defining the Optimal Region of Interest for Hyperemia Grading in the Bulbar Conjunctiva

**DOI:** 10.1155/2016/3695014

**Published:** 2016-12-19

**Authors:** María Luisa Sánchez Brea, Noelia Barreira Rodríguez, Antonio Mosquera González, Katharine Evans, Hugo Pena-Verdeal

**Affiliations:** ^1^VARPA Group, Department of Computer Science, University of A Coruna, A Coruna, Spain; ^2^Artificial Vision Group, Department of Electronics and Computer Science, University of Santiago de Compostela, Santiago de Compostela, Spain; ^3^School of Optometry and Vision Sciences, Cardiff University, Wales, UK; ^4^Optometry Group, Department of Applied Physics, University of Santiago de Compostela, Santiago de Compostela, Spain

## Abstract

Conjunctival hyperemia or conjunctival redness is a symptom that can be associated with a broad group of ocular diseases. Its levels of severity are represented by standard photographic charts that are visually compared with the patient's eye. This way, the hyperemia diagnosis becomes a nonrepeatable task that depends on the experience of the grader. To solve this problem, we have proposed a computer-aided methodology that comprises three main stages: the segmentation of the conjunctiva, the extraction of features in this region based on colour and the presence of blood vessels, and, finally, the transformation of these features into grading scale values by means of regression techniques. However, the conjunctival segmentation can be slightly inaccurate mainly due to illumination issues. In this work, we analyse the relevance of different features with respect to their location within the conjunctiva in order to delimit a reliable region of interest for the grading. The results show that the automatic procedure behaves like an expert using only a limited region of interest within the conjunctiva.

## 1. Introduction

Hyperemia is the occurrence of an engorgement in a blood vessel. As the blood accumulates, a characteristic red colouration appears in the surrounding area. When the affected tissue is the bulbar conjunctiva, we refer to it as bulbar hyperemia. Bulbar hyperemia can appear due to normal bodily processes, but it can also serve as an indicator of the first stages of some pathologies, such as dry eye syndrome or allergic conjunctivitis. These pathologies have a high incidence in the world population and, more importantly, they have a growing prevalence. Therefore, hyperemia grading is crucial to the prompt detection of these health problems and, therefore, has both medical and economical repercussions.

**Figure 1:**
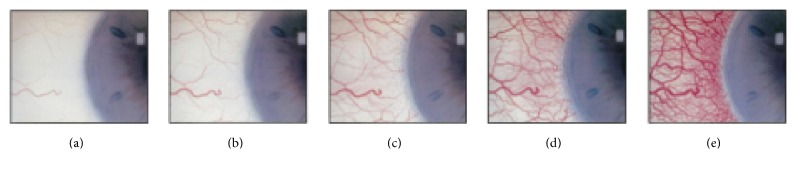
Efron grading scale. From (a) to (e), lower to higher values.

The manual process that optometrists have to face is tedious, time consuming, highly subjective, and nonrepeatable. The first step is to obtain a video or picture of the patient eye. Then, the image or images must be analysed in detail, searching for indicators of the symptom, such as the aforementioned red hue. Finally, the optometrist compares the patient's eye with a given grading scale, in order to obtain the final evaluation. Grading scales are collections of images that show the different levels of severity that bulbar hyperemia can present. One of the most widely used is Efron grading scale, which consists of four images labelled from 0 to 4 as depicted in Figure 1. Level 0 represents a perfectly white eye, while level 4 indicates a severe health problem. The specialists have to look for the grade of the scale that is the most similar to the patient and, additionally, they have to measure the difference between the patient and the prototype. This is because the evaluation is represented by a number with a decimal part, as four or five values are not enough to represent the symptoms accurately.

All of the drawbacks in the manual process can be solved by the automation of the process. We developed a fully automatic methodology for bulbar hyperemia grading that comprises three steps: the segmentation of the region of interest within the bulbar conjunctiva, the computation of several hyperemia indicators, and, finally, the transformation of the computed features to the grade in a grading scale.

**Figure 2:**
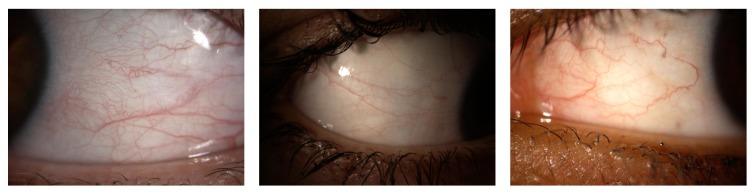
Different image conditions mainly due to illumination issues and the position of the eye.

Regarding the first step of the methodology, obtaining an accurate segmentation of the conjunctiva has proven to be a far from straightforward task. The main problem is the variability of the images, including but not limited to a wide spectrum of illumination conditions, the location of the eye in the image, the devices used to take the pictures or videos, the distance from the eye to the camera, or the presence of eyelashes. Examples of this variability are shown in Figure 2.

As a consequence, the segmentation of the whole conjunctiva is not straightforward and entails a high computational cost. However, although specialists look at the whole area when performing the grading, it is not proven that they use it evenly. As knowledge is difficult to model even for the experts themselves, we decided to study the effects of restricting the computation of the hyperemia features to the central area of the picture. The approach of using only a part of the image is supported by works such as [1], where a rectangle is manually selected in the image in order to define the region of interest for a comparison between objective and subjective methods. In [2], a rectangular region of interest is also defined in the image. The authors analyse the influence of the number of vessels in hyperemia, but they do not perform a further assessment.

In this work we analyse the results of several segmentation algorithms in the conjunctival area and we study the influence of different regions of interest in the computation of the hyperemia grading value. To this end, we compute several features of interest in these regions based on colour and the presence of vessels and we analyse their contribution to the final value by means of feature selection techniques. Finally, we use regression methods to transform the selected feature vectors to a more suitable representation in a grading scale.

This work is structured as follows.  Section 2 explains the methodology for conjunctival segmentation and feature computation.  Section 3 shows the results of the proposed methodology. Finally, Section 4 presents the conclusions and future work.

## 2. Methodology

Our methodology for hyperemia grading can be divided into two distinctive parts: on the one hand, the extraction of a set of features from a region of interest by means of image processing algorithms and on the other hand, the transformation of these features into values in a grading scale using regression techniques. The former comprises the detection of the region of interest and the computation of features from the image pixels whereas the later requires the selection of the most representative features, the creation of suitable training and testing datasets, and the evaluation of several regression algorithms.

In this section, we analyse our dataset in order to select an appropriate subset of images and grading for the study. Then, we propose several segmentation algorithms to detect the conjunctiva in these images. Finally, we introduce the features that are computed in the region of interest.

### 2.1. Data Preparation

Our image set consists of 141 images of the bulbar conjunctiva. The images show a side view of the eye, from the pupil to one of its opposite corners (lacrimal area or corner of the eye area). There are images from both eyes and both side views of the eye. The images were obtained with a slit lamp camera (Bon 75-SL DigiPro3 HD, Bonn, Germany) in the School of Optometry and Vision Sciences at the Cardiff University. The image resolution is 1600 × 1200 px.

**Figure 3:**
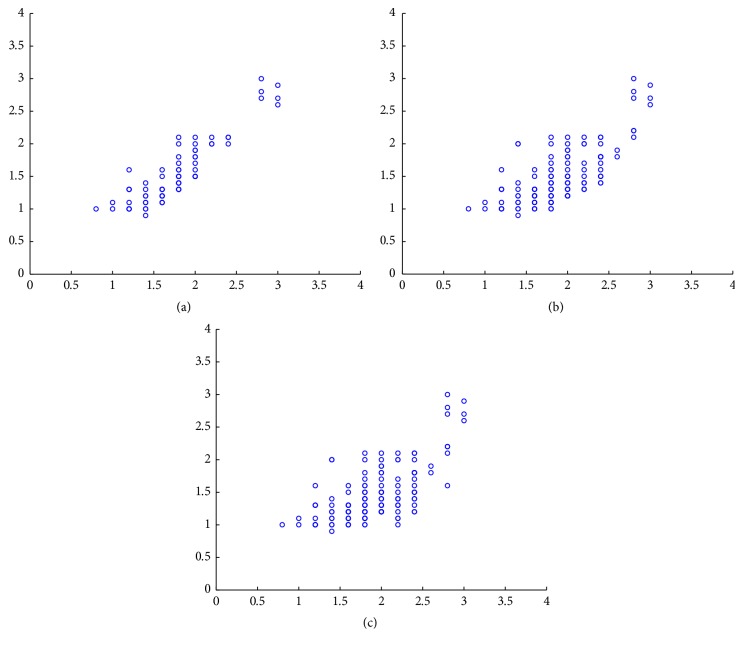
Correlation between the experts' evaluation. Each axis shows one of the expert's gradings. (a) to (c): threshold = 0.5, threshold = 1.0, and threshold = 1.5.

Two optometrists evaluated the whole image set using the Efron grading scale in a blinded manner and they did not communicate with each other during the process. The correlation of their gradings was 0.66, which can be considered a good correlation for this kind of scenario, but not enough for machine learning techniques. Therefore, we decided to refine the image set by removing those images where the difference between the evaluations was above a given threshold.  Table 1 shows the evolution of the correlation with respect to several threshold values as well as the number of remaining images. Additionally, Figure 3 shows the distribution of the gradings in a scatter plot.

In view of the data, our final dataset consists of 76 images where the experts' evaluations differ less than 0.5 points. This reduced image set has a correlation of almost 0.9. We use the average value of the two evaluations as our ground truth for the machine learning algorithms.

### 2.2. Extraction of the Regions of Interest

The white part of the conjunctiva is the region where the experts focus their attention for hyperemia grading. Thus, its location is the first step in our methodology. We explore several approaches in order to study the influence of the region of interest in the final grading value.

First, we tested several state-of-the-art methods in order to automatically segment the conjunctiva:

(i) *T*_*G*_: thresholding in the green channel of the RGB image, using an average of the intensity in the whole image.
(ii) *T*_*G*′_: thresholding in the green channel of the RGB image, using an average of the intensity of the pixels in the central horizontal stripe of the image.
(iii) *T*_*S*_: thresholding in the *S* channel of the TSL image.
(iv) *T*_*S*′_: thresholding in the *S* channel of the TSL image, correcting the level of red to remove the vessel influence in the more severe hyperemia images.
(v) *T*_*V*_: thresholding in the *V* channel of the HSV image.
(vi) *T*_*a*_: thresholding in the a-channel of the *L* ^*∗*^ *a* ^*∗*^ *b* ^*∗*^ image.
(vii) *O*_*W*_: watershed segmentation.
(viii) *O*_SM_: split-and-merge segmentation.

**Figure 4:**
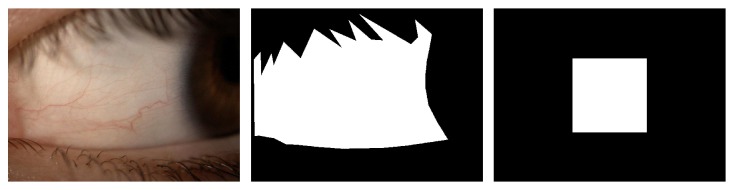
Conjunctiva image, manual segmentation of the region of interest, and central square of 512 × 512 px.

We segmented our dataset of 76 images manually in order to ensure that the segmentation of the region of interest does not influence the computation of the features. To this end, we use the function* roipoly* from MATLAB [3] that allow us to manually define the vertices of a polygonal mask in the input image. Then, we select a square of 512 × 512 px in the centre of this mask, as depicted in Figure 4. The images used for hyperemia grading are centred in the conjunctival area so that the iris and the corner of the eye are always placed near the image boundaries. This way, a centred region is mostly composed of conjunctival pixels. Moreover, the iris, eyelids, and eyelashes are removed by means of the manual mask, so they will not add bias in the results even if they are within the scope of the rectangle.

We decided to use this region because larger regions are not available in all the images due to the position of the eye within the image and the variability regarding the position of the eyelids. Moreover, we are interested in comparing the regions that are present in all the images, which are only the most centred. Regarding the size of the area, previous works in the literature support that even smaller rectangle sizes are significant enough for the grading [2].

Most of the images of our data set show a close view of the conjunctiva, with the eye fully open and small eyelid areas. However, there were 6 images that presented the eye much more closed and the conjunctiva was too small to produce a 512 × 512 px square, leading us to discard those images.

We divided this central square into cells. Among the many grid possibilities, we decided to test 1 × 2, 2 × 1, and 2 × 2 grids, as we considered that a region smaller than 256 × 256 was too small to provide a useful approach to the measurement.

The results of a previous study confirmed that there are differences between the pupil area and the opposite side of the eye [4]. Since we are interested in the same cells showing the same areas of the eye, we flipped vertically some of the images. Thus, all of them had the pupil in the same side.

### 2.3. Feature Computation

In previous works [5], we studied the features that best represent the bulbar conjunctival hyperemia. We apply these features to each of the cells, the whole squared region of interest, and the whole manual segmentation of the conjunctiva. Hence, we obtain a feature vector of (*m∗n* + 2)*∗f* values for each *m* × *n* grid, where *f* is the number of features.  Table 2 summarises the 24 features computed.

In the equations, *n* and *m* indicate the size of the input image *I*, but considering only the pixels that belong to the region of interest; *i* and *j* represent the position (row, column) of the current pixel in the image; *R*, *G*, and *B* indicate the channel value in RGB colour space; *H*, *S*, and *V* represent the channel value in HSV colour space; *L*, *a*, and *b* represent the channel value in *L* ^*∗*^ *a* ^*∗*^ *b* ^*∗*^ colour space; *E* is the edge image; *VE* is the set of vessel edges within the region of interest, whereas $\bar{VE}$ are the nonvessel pixels. The vessel edges are computed using the Canny edge detector. In the feature *F*_1_, *n*_*r*_ is the number of image rows considered, and *M* is a mask. The feature *F*_13_ computes the red hue value taking into account the values of the neighbouring pixels. *μ*_*ij*_ is the value for this neighbourhood.

Each value of the feature vector is denoted with the feature number and a subscript that represents the region where the feature is computed. This way, subscript *o* represents that the feature is computed in the whole conjunctiva; subscript *t*, in the 512 × 512 square, and subscript *g* plus a number indicate the cell grid number. The cells are numbered as (*row*, *column*), with the relative position in the original square.

After we have computed the 24 features in each region of interest, we have a feature vector with different ranges of values in each cell. We need to transform these values to a grade within the scale range by means of a complex function, as there is no apparent relationship between the values and the final grade. As a previous step to the transformation to the grading scale, we are interested in using only the values of the most relevant features in the computation. To that end, we use feature selection methods. We had previously analysed several feature selection techniques in order to reduce the dimensionality of the problem [6]. In this article, we apply the method that provided the best results for the Efron scale, the filter method Correlation Feature Selection (CFS) [7]. However, in order to further analyse how the best features are selected in the grid configurations, we added the filter ranker method Relief [8] and the wrapper method SMOReg [9].

We also performed a comparison of different machine learning techniques [4]. The one that obtained the best results was the Multilayer Perceptron (MLP) [10] approach, followed by the Partial Least Square (PLS) regression [11] and the Random Forest (RF) [12]. Therefore, we implemented these three approaches, as each of them belongs to a different type and, hence, we can compare their behaviour with the set of regions of interest.

## 3. Results

In this section we present the results of the proposed segmentation approaches and we study the relevance of the features computed in different regions of interest. Finally, we test the best combinations of features with several regression techniques in order to emulate the experts' gradings.

First, we compared the manual segmentation of the images with the automatic approaches by computing the number of true positives (both methods identify the pixel as part of the conjunctiva), false positives (the automatic method identifies background as conjunctiva), true negatives (both methods identify a pixel as background), and false negatives (the automatic method identifies a part of the conjunctiva as background). Then, we computed the specificity, sensitivity, accuracy, and precision of each method. All the segmentation methods were implemented in C++ with the OpenCV library [13].  Table 3 depicts the results for the state-of-the-art conjunctiva segmentation techniques. Despite obtaining some high values for the parameters, the main drawback of these approaches is that they do not provide acceptable values for all parameters at a time. Some of the approaches are too inclusive, while others remove a large part of the conjunctiva.

We consider desirable that all the parameters are, at least, at 80%. Split-and-merge segmentation, while close to this requirement, is computationally costly. The computation takes more than 6 seconds on average, while thresholding approaches take less than a second on the same computer.

**Figure 5:**
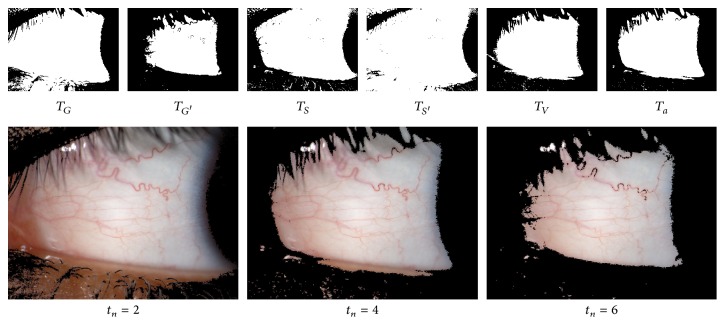
Individual thresholding masks and the resulting combinations with different values of *t*_*n*_.

**Figure 6:**
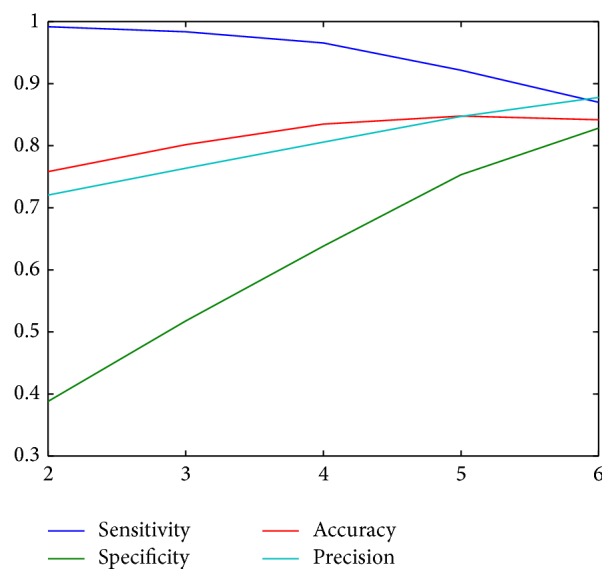
Sensitivity, specificity, accuracy, and precision for the threshold combinations.

Therefore, we decided to perform a test combining all the proposed thresholding approaches. We threshold the input image with the six aforementioned intensity threshold values. The pixels over the threshold at least *t*_*n*_ times are considered part of the conjunctiva and the remaining pixels are marked as background; that is, we obtain six different segmentations for an image and we create the final mask by using only the pixels that belong to at least *t*_*n*_ of the masks. We tested this approach with *t*_*n*_ ranging from 2 to 6 (Figure 5). The results for the different threshold approaches are depicted in Figure 6. We can see that the optimal value for the dataset is 6 since all the statistical measures are above 0.8.

Since a precise segmentation of the conjunctiva is hard to obtain, we analyse if a smaller region is enough to develop an automatic grading system. To this end, we study the relevance of each feature in several regions of interest defined within the conjunctiva.

In order to discover which areas are the most relevant for the evaluation, we computed the hyperemia image features for the 70 images, obtaining one feature vector for each configuration grid, 1 × 2, 2 × 1, and 2 × 2. Next, we applied the feature selection techniques. We used a 10-fold cross-validation and we averaged the occurrences of the features among the folds in order to decide the final subset. For CFS and SMOReg, we selected those features that were selected in at least 7 out of 10 folds. The ranker method is slightly different, as it always return all the features, but sorted in descending order of importance. Hence, we decided to take into account only the features that, on average, were selected on the first 10 positions of the ranking.  Table 4 shows the features selected for each grid and method.

We can observe how the methods favour the larger areas (central square and full conjunctiva). This was expected, as they provide more information than the cells. However, there are a few exceptions such as feature 23 (white in background, HSV colour space) in CFS, feature 15 (a-channel in vessels, *L* ^*∗*^ *a* ^*∗*^ *b* ^*∗*^ colour space) in Relief, or feature 17 (yellow in background, HSV colour space) in SMOReg. This leads us to think that there are, in fact, some areas where a feature can be specially representative.

Table 5 depicts the mean square error (MSE) values for each combination of grid, feature selection method, and machine learning technique. We also include the results of the whole conjunctiva manually segmented. The best value for configurations 1 × 2 and 2 × 1 is achieved by the MLP with all the features. For the last configuration, 2 × 2, the best value is obtained also by the MLP, but using the SMOReg subset: only feature 14 (a-channel of the image, *L* ^*∗*^ *a* ^*∗*^ *b* ^*∗*^ colour space) computed in the whole conjunctiva.

With these experiments, we notice how, despite most features belonging to the larger areas of the image, some of the features are selected as relevant in the individual cells. This leads us to question if we are able to evaluate the hyperemia grade taking into account only the individual cells. Therefore, we performed feature selection with only the features computed from the cells and applied the regression techniques to the obtained subsets. The selected features are depicted in Table 6.

In view of the data, we notice that some of the most common features, such as feature 10 and feature 23, remain being favoured by the feature selection techniques. However, the most common one, feature 14, does not appear in none of CFS subsets, nor in most of SMOReg ones. This gives us the idea that the a-channel of the image (red hue level in *L* ^*∗*^ *a* ^*∗*^ *b* ^*∗*^ colour space) is highly relevant when it takes place in a large area, but not when we limit the region size. On the other side, feature 12 (percentage of red, HSV colour space) now appears in two of the SMOReg subsets.

Regarding the cell importance, in 1 × 2 and 2 × 1 configurations, filter methods seem to pick evenly features in both areas. SMOReg, on the contrary, favours the left and bottom areas of the eye. This remains true for the last configuration, 2 × 2, where it selects only features in the lower left corner. Finally, the filters choose all cells but (1,1) at least once in the 2 × 2 scenario, which lead us to think that the upper left corner is the less relevant part.

The MSE results for each situation are shown in Table 7. Again, the best value for the 1 × 2 configuration is achieved by the MLP with all the features. What is interesting in this value is that it improves the original minimal MSE obtained by using the features in the whole conjunctiva and in the central square. The best values for the 2 × 1 and 2 × 2 configurations are obtained by the PLS approach with the Relief subset and the MLP approach with the CFS subset, respectively. In these cases, we do not improve the previous minimum value for the given division, but we still obtain an error value lower than 0.1. As we mentioned when analysing the correlation values, it is not uncommon that the experts obtain differences in evaluation higher than 0.5, this is, squared errors higher than 0.25. Consequently, we can affirm that our system is able to behave like a human expert taking into account a reduced region of interest.

## 4. Conclusions

In this paper, we use our fully automatic hyperemia grading framework in order to identify the most relevant areas of interest in the bulbar conjunctiva. There were two main reasons for this experiment. First, we wanted to know if a smaller area of the conjunctiva is representative enough for grading purposes since the segmentation of the conjunctiva is still an open task. Second, we aimed to identify the areas where a feature is more important, and the areas that have most of the specialists' attention. Thus, we selected the central square of the image because it is the more constant section among the pictures, as it is present when the eye is half closed or the camera is moved to the left or right sides. We also subdivided the square into cells in order to test even smaller areas. To this end, we apply several feature selection methods and results show that some features are indicators of hyperemia even if they only take place in a section of the conjunctiva. We applied three regression techniques in order to transform the feature vectors computed in different regions of interest into the grading scale values. When using both global (whole conjunctiva and central square) and cell features, the best MSE result was obtained by the MLP with all the features in the 1 × 2 grid configuration. However, this value is improved by using only the features in the cells. In fact, several combinations of grids, feature selection subsets, and regression techniques obtain lower error results by using some of the features computed in only a part of the image. Therefore, we can conclude that we can use only the central area of the image when aiming for the segmentation of the bulbar conjunctiva as it is representative enough. This translates in a reduction of the computational time and a lower chance of including unwanted information within the region of interest, such as eyelids or eyelashes. Also, the test performed with only the features computed in the cells gives us the idea that the lower region of the eye is more important, and so it is the pupil side.

Our future lines of work include the development of an application for the automatic evaluation of bulbar hyperemia and the subsequent integration of these results in the final methodology.

## Figures and Tables

**Table 1:** Correlation between the experts' evaluations.

Threshold	# images	Correlation
0.5	76	0.8981
1.0	133	0.7212
1.5	141	0.6609

**Table 2:** Implemented hyperemia features.

Feature	Name	Formula
*F*_1_	Vessel count	$\frac{\sum_{i=1}^{n}\sum_{j=1}^{m}E_{ij}M_{ij}}{n_{r}}$
*F*_2_	Vessel occupied area	$\frac{\sum_{i=1}^{n}\sum_{j=1}^{m}VE_{ij}}{nm}$
*F*_3_	Relative vessel redness	$\;\overset{n}{\underset{i=1}{\sum }}\overset{m}{\underset{j=1}{\sum }}\left(\frac{R_{ij}VE_{ij}}{R_{ij}+G_{ij}+B_{ij}}\right)$
*F*_4_	Relative image redness	$\;\overset{n}{\underset{i=1}{\sum }}\overset{m}{\underset{j=1}{\sum }}\left(\frac{R_{ij}}{R_{ij}+G_{ij}+B_{ij}}\right)$
*F*_5_	Difference red-green in vessels	$\frac{\sum_{i=1}^{n}\sum_{j=1}^{m}\left(\left(R_{ij}-G_{ij}\right)VE_{ij}\right)}{nm}$
*F*_6_	Difference red-green of the image	$\frac{\sum_{i=1}^{n}\sum_{j=1}^{m}\left(R_{ij}-G_{ij}\right)}{nm}$
*F*_7_	Difference red-blue in vessels	$\frac{\sum_{i=1}^{n}\sum_{j=1}^{m}\left(\left(R_{ij}-B_{ij}\right)VE_{ij}\right)}{nm}$
*F*_8_	Difference red-blue of the image	$\frac{\sum_{i=1}^{n}\sum_{j=1}^{m}\left(R_{ij}-B_{ij}\right)}{nm}$
*F*_9_	Red hue value	$\frac{\sum_{i=1}^{n}\sum_{j=1}^{m}\left|128-H_{ij}\right|}{nm}$
*F*_10_	Percentage of vessels	$\frac{\sum_{i=1}^{n}\sum_{j=1}^{m}VE_{ij}}{nm}100$
*F*_11_	Percentage of red (RGB)	$\frac{\sum_{i=1}^{n}\sum_{j=1}^{m}R_{ij}VE_{ij}}{\sum_{i=1}^{n}\sum_{j=1}^{m}VE_{ij}}100$
*F*_12_	Percentage of red (HSV)	$\frac{\sum_{i=1}^{n}\sum_{j=1}^{m}H_{ij}VE_{ij}}{\sum_{i=1}^{n}\sum_{j=1}^{m}VE_{ij}}100$
*F*_13_	Redness with neighbourhood	$\;\overset{n}{\underset{i=1}{\sum }}\overset{m}{\underset{j=1}{\sum }}\frac{H_{ij}VE_{ij}}{\mu_{ij}}$
*F*_14_	*L* ^*∗*^ *a* ^*∗*^ *b* ^*∗*^ *a*-channel of the image	$\frac{\sum_{i=1}^{n}\sum_{j=1}^{m}a_{ij}}{nm}$
*F*_15_	*L* ^*∗*^ *a* ^*∗*^ *b* ^*∗*^ *a*-channel in vessels	$\frac{\sum_{i=1}^{n}\sum_{j=1}^{m}\left(a_{ij}VE_{ij}\right)}{nm}$
*F*_16_	Yellow in background (RGB)	$\frac{\sum_{i=1}^{n}\sum_{j=1}^{m}\left(\left(R_{ij}+G_{ij}\right){\bar{VE}}_{ij}\right)}{nm}$
*F*_17_	Yellow in background (HSV)	$\frac{\sum_{i=1}^{n}\sum_{j=1}^{m}\left(\left|240-H_{ij}\right|{\overset{-}{VE}}_{ij}\right)}{nm}$
*F*_18_	Yellow in background (*L* ^*∗*^ *a* ^*∗*^ *b* ^*∗*^)	$\frac{\sum_{i=1}^{n}\sum_{j=1}^{m}\left(b_{ij}{\bar{VE}}_{ij}\right)}{nm}$
*F*_19_	Red in background (RGB)	$\frac{\sum_{i=1}^{n}\sum_{j=1}^{m}\left(R_{ij}{\bar{VE}}_{ij}\right)}{nm}$
*F*_20_	Red in background (HSV)	$\frac{\sum_{i=1}^{n}\sum_{j=1}^{m}\left(\left|128-H_{ij}\right|{\bar{VE}}_{ij}\right)}{nm}$
*F*_21_	Red in background (*L* ^*∗*^ *a* ^*∗*^ *b* ^*∗*^)	$\frac{\sum_{i=1}^{n}\sum_{j=1}^{m}\left(a_{ij}{\bar{VE}}_{ij}\right)}{nm}$
*F*_22_	White in background (RGB)	$\frac{\sum_{i=1}^{n}\sum_{j=1}^{m}\left(\left(R_{ij}+G_{ij}+B_{ij}\right){\bar{VE}}_{ij}\right)}{nm}$
*F*_23_	White in background (HSV)	$\frac{\sum_{i=1}^{n}\sum_{j=1}^{m}\left(\left(V_{ij}+S_{ij}\right){\bar{VE}}_{ij}\right)}{nm}$
*F*_24_	White in background (*L* ^*∗*^ *a* ^*∗*^ *b* ^*∗*^)	$\frac{\sum_{i=1}^{n}\sum_{j=1}^{m}\left(L_{ij}{\bar{VE}}_{ij}\right)}{nm}$

**Table 3:** Sensitivity, specificity, accuracy, and precision for each ROI extraction procedure.

Mask	Sensitivity	Specificity	Accuracy	Precision
*T*_*G*_	0.895698	0.65153	0.798084	0.810654
*T*_*G*′_	0.618007	0.97758	0.746112	0.975355
*T*_*S*_	0.909963	0.750125	0.841281	0.846001
*T*_*S*′_	0.959717	0.452372	0.760782	0.737163
*T*_*V*_	0.776801	0.848024	0.787575	0.87764
*T*_*a*_	0.795949	0.895013	0.817989	0.910256

*O*_*W*_	0.947957	0.352172	0.722338	0.709184
*O*_SM_	0.797081	0.9071	0.82936	0.924299

**Table 4:** Features chosen with each division and feature selection method.

Grid	
	CFS
1 × 2	*F*_2*o*_, *F*_10*o*_, *F*_14*o*_, *F*_23*o*_, *F*_10*t*_, *F*_23*t*_, *F*_23*g*1_, *F*_2*g*2_
2 × 1	*F*_2*o*_, *F*_10*o*_, *F*_14*o*_, *F*_10*t*_, *F*_23*t*_, *F*_23*g*1_, *F*_23*g*2_
2 × 2	*F*_2*o*_, *F*_10*o*_, *F*_14*o*_, *F*_10*t*_, *F*_23*t*_, *F*_23*g*11_, *F*_23*g*21_, *F*_2*g*22_

	Relief
1 × 2	*F*_14*o*_, *F*_21*o*_, *F*_15*o*_, *F*_5*o*_, *F*_6*o*_, *F*_7*o*_, *F*_5*t*_
2 × 1	*F*_14*o*_, *F*_21*o*_, *F*_15*o*_, *F*_5*o*_, *F*_6*o*_, *F*_5*t*_, *F*_15*g*2_, *F*_7*o*_
2 × 2	*F*_14*o*_, *F*_21*o*_, *F*_15*o*_, *F*_5*o*_, *F*_6*o*_, *F*_7*o*_, *F*_5*t*_, *F*_11*o*_

	SMOReg
1 × 2	*F*_10*o*_, *F*_14*o*_
2 × 1	*F*_14*o*_, *F*_17*g*2_
2 × 2	*F*_14*o*_

**Table 5:** MSE values for each combination of grid, feature selection method, and machine learning technique.

Grid	All	CFS	Relief	SMOReg
	MLP			
1 × 2	**0.02214**	0.22139	0.04798	0.04467
2 × 1	**0.03009**	0.03854	0.04552	0.05429
2 × 2	0.22129	0.04511	0.03049	**0.03048**
Conjunctiva	0.22148	0.22293	0.22131	0.05735

	PLS			
1 × 2	0.07173	0.08799	0.05257	0.05313
2 × 1	0.05846	0.11388	0.05417	0.06370
2 × 2	0.07172	0.14042	0.05242	0.06077
Conjunctiva	0.06432	**0.05307**	0.05470	0.05354

	RF			
1 × 2	0.08297	0.07985	0.09575	0.07868
2 × 1	0.08993	0.08097	0.09308	0.10824
2 × 2	0.08635	0.08042	0.09224	0.10231
Conjunctiva	0.08338	0.10235	0.09734	0.10887

**Table 6:** Features chosen with each grid and feature selection method (cells only).

Grid	
	CFS
1 × 2	*F*_10*g*1_, *F*_23*g*1_, *F*_2*g*2_, *F*_10*g*2_
2 × 1	*F*_10*g*1_, *F*_23*g*1_, *F*_5*g*2_, *F*_10*g*2_, *F*_15*g*2_
2 × 2	*F*_10*g*12_, *F*_2*g*21_, *F*_10*g*21_, *F*_15*g*21_, *F*_23*g*21_, *F*_2*g*22_, *F*_10*g*22_

	Relief
1 × 2	*F*_14*g*1_, *F*_5*g*1_, *F*_14*g*2_, *F*_21*g*1_, *F*_7*g*1_, *F*_5*g*2_, *F*_6*g*1_, *F*_21*g*2_, *F*_15*g*1_, *F*_6*g*2_, *F*_7*g*2_, *F*_15*g*2_
2 × 1	*F*_15*g*2_, *F*_14*g*2_, *F*_21*g*2_, *F*_7*g*2_, *F*_6*g*2_, *F*_5*g*1_, *F*_8*g*2_, *F*_7*g*1_, *F*_15*g*1_, *F*_18*g*2_
2 × 2	*F*_14*g*21_, *F*_5*g*21_, *F*_21*g*21_, *F*_7*g*21_, *F*_6*g*21_, *F*_14*g*12_, *F*_15*g*21_, *F*_21*g*12_, *F*_8*g*21_, *F*_14*g*22_, *F*_6*g*12_

	SMOReg
1 × 2	*F*_12*g*1_
2 × 1	*F*_15*g*2_
2 × 2	*F*_12*g*21_, *F*_14*g*21_

**Table 7:** MSE values for each combination of grid, feature selection method, and machine learning technique (cells only).

Grid	All	CFS	Relief	SMOReg
	MLP			
1 × 2	**0.02148**	0.10003	0.22129	0.10230
2 × 1	0.22143	0.22129	0.22135	0.35284
2 × 2	0.22140	**0.05455**	0.22136	0.05779

	PLS			
1 × 2	0.07135	0.11707	0.08559	0.23905
2 × 1	0.06881	2.93832	**0.06830**	0.06981
2 × 2	0.09540	0.09608	0.07756	0.07056

	RF			
1 × 2	0.09263	0.14607	0.09993	0.25122
2 × 1	0.09951	0.09954	0.10945	0.10790
2 × 2	0.10317	0.11226	0.10531	0.12962
